# Mitochondrial DNA oxidation and content in different metabolic phenotypes of women with polycystic ovary syndrome

**DOI:** 10.3389/fendo.2024.1501306

**Published:** 2025-01-09

**Authors:** Mailén Rojo, Hernán Pérez, Andrea Liliana Millán, María Constanza Pautasso, Alejandra Duarte, Giselle Adriana Abruzzese, Alicia Beatriz Motta, Gustavo Daniel Frechtel, Gloria Edith Cerrone

**Affiliations:** ^1^ Facultad de Farmacia y Bioquímica, Departamento de Microbiología, Inmunología, Biotecnología y Genética, Universidad de Buenos Aires, Buenos Aires, Argentina; ^2^ Instituto de Inmunología, Genética y Metabolismo (INIGEM), Universidad de Buenos Aires – National Scientific and Technical Research Council (CONICET), Buenos Aires, Argentina; ^3^ Hospital de Clínicas José de San Martin, Servicio de Nutrición, Buenos Aires, Argentina; ^4^ Fundación Héctor Alejandro (H.A.) Barceló, Instituto Universitario de Ciencias de la Salud, Buenos Aires, Argentina; ^5^ Centro de Estudios Farmacológicos y Botánicos (CEFYBO), Laboratorio de Fisiopatología ovárica, Universidad de Buenos Aires – National Scientific and Technical Research Council (CONICET), Buenos Aires, Argentina

**Keywords:** obesity, oxidative damage, metabolic syndrome, polycystic ovary syndrome, mitochondrial DNA

## Abstract

**Introduction:**

Polycystic Ovary Syndrome (PCOS) affects 5-20% of reproductive-aged women. Insulin resistance (IR) is common in PCOS with consequent elevated risks of metabolic disorders and cardiovascular mortality. PCOS and obesity are complex conditions associated with Metabolic Syndrome (MS), contributing to cardiovascular disease and type 2 diabetes mellitus (T2D). Obesity and PCOS exacerbate each other, with central obesity driving metabolic changes. Mitochondrial dysfunction, characterized by oxidative stress and reduced antioxidant capacity, plays a key role in PCOS pathology.

**Methods:**

In our study, we investigated 81 women with PCOS, and 57 control women aged 16 to 46 years old. Relative mitochondrial DNA (mtDNA) content and its oxidation level (8-oxoguanine, 8-OxoG) were determined in peripheral blood leukocytes by the SYBR Green method real-time PCR.

**Results:**

Our findings showed that patients with PCOS had decreased mtDNA content and increased oxidation damage. Stratifying these patients by metabolic profile, revealed a progressive decline in mtDNA content from the normal-weight control group to the MHO-PCOS and MUO-PCOS groups, suggesting that lower mtDNA content is linked to obesity and worse metabolic profile. However, mtDNA oxidation levels did not differ significantly among these groups. Additionally, the decline in mtDNA content and the increase in oxidation levels between controls and patients with PCOS lost significance when these relationships were adjusted for the HOMA index.

**Discussion:**

This finding suggests that IR could be the main factor contributing to mitochondrial dysfunction in PCOS. Maintaining optimal mtDNA copies are crucial for mitochondrial and cell function, suggesting potential therapeutic targets for PCOS-associated metabolic disturbances.

## Introduction

Polycystic ovary syndrome (PCOS) affects 5-20% of women of reproductive age (1) and is characterized by chronic menstrual disorders, hirsutism, acne, hyperinsulinemia, and infertility. PCOS has been further linked to anovulation, mitochondrial dysfunction, and hormonal imbalance (2). Eighty percent of patients with PCOS present obesity, insulin resistance (IR), and higher frequency of glucose intolerance (35-40%) and/or diabetes (7.5-10%) than the general population at an early age. Patients with PCOS with IR and/or type 2 diabetes mellitus (T2D) have a 5-fold increase in cardiovascular mortality rates. PCOS and obesity are polygenic and multifactorial entities that may be associated with features of metabolic syndrome (MS), which constitutes the leading cause of T2D development and cardiovascular morbidity and mortality (3).

Due to its phenotypic heterogeneity, the diagnosis of PCOS is complex. According to the Rotterdam consensus of 2003 (4), PCOS is diagnosed in patients who meet at least two of the following criteria: clinical or biochemical hyperandrogenism, oligo- or amenorrhea, and polycystic ovary morphology. A bidirectional relationship has been documented between obesity and PCOS, with both exacerbating each other in a synergic manner (5). The genetic-molecular basis linking these pathologies remains to be established. Obesity, particularly visceral obesity, is chiefly responsible for metabolic alterations. In addition, obesity increases IR and triggers compensatory hyperinsulinemia, which in turn increases adipogenesis and reduces lipolysis. Hyperinsulinemia also influences thecal cell sensitivity to luteinizing hormone (LH) stimulation, which positively regulates the production of ovarian androgens with consequent hyperandrogenism (6).

Mitochondria play a critical role in cellular homeostasis by regulating processes such as apoptosis, mitosis, differentiation, and survival. Impaired mitochondrial function leads to oxidative phosphorylation inhibition and increased reactive oxygen species (ROS) production, which boosts oxidative stress, damages macromolecules, and eventually affects cellular homeostasis (7). PCOS is associated with an increase in oxidative stress and a decrease in antioxidant concentrations, in mitochondrial membrane potential, in glutathione levels (indicative of increased ROS production), and in ATP production (8). High levels of free radicals can cause alterations in mtDNA, leading to a reduction in mitochondrial content and function over time. In this context, mtDNA content and oxidation levels may help determine mitochondrial dysfunction and damage caused by oxidative stress, thus emerging as potential biomarkers for metabolic alterations in PCOS (9). In previous studies by our group, a shorter average telomere length (aTL) was found to be potentially related to the presence of obesity in PCOS. This finding aligns with evidence suggesting that mitochondrial function is also adversely affected by obesity, as both conditions are associated with increased oxidative stress and metabolic dysfunction (10).

The metabolically healthy obese patients (MHO) are a subgroup of individuals who are not affected by MS and account for 10%-40% of the obese population depending on the population under study and the classification criteria (11, 12). MHO have lower risk of mortality, progression to cardiovascular disease, and progression to T2D than metabolic unhealthy obese patients (MUO) (13, 14). While the MHO phenotype is well documented among obese individuals, both male and female, evidence of MHO and MUO subtypes in obese women with PCOS remains scarce. Kim et al. studied 70 obese girls with PCOS from the PCOS Center at the Children’s Hospital of Pittsburgh and classified them into 19 MHO and 51 MUO based on insulin sensitivity. The study found that MHO-PCOS had a lower risk of T2D, atherogenesis, inflammation, and hyperandrogenemia than MUO-PCOS (15). In turn, Liang et al. examined Chinese women from the Shanghai region, classifying 112 as MHO and 299 as MUO. The authors reported a similar prevalence of PCOS between groups but a better metabolic profile in MHO, with less visceral adipose tissue and higher insulin sensitivity (16). Mu et al. also conducted a survey in China and observed higher risk of PCOS and chronic anovulation in MHO women, with obesity as a potential independent risk factor (17). Finally, Barrea et al. characterized the endocrine-metabolic profile and inflammatory status of 54 MHO-PCOS and 40 patients with MUO-PCOS in Naples, Italy, and highlighted the utility of cardio-metabolic indices (Visceral Adiposity Index and Fatty Liver Index) for distinguishing MUO and MHO phenotypes in PCOS (18).

In a previous study, we analyzed mtDNA content and oxidation in MHO and MUO phenotypes in patients without PCOS, providing important context on the relationship between mitochondrial parameters, oxidative stress, and metabolic status. This led us to hypothesize that metabolic status might be contributing to the mitochondrial alterations observed (19). In the current study, we extended the research to women with PCOS, comparing them to a normal weight control group. The etiology of PCOS remains unknown, highlighting the need for identifying parameters that can better characterize the syndrome and its subgroups. This study is significant in defining new factors associated with the varied metabolic states of patients, offering potential insights into more personalized approaches to understanding and managing PCOS. In this context, this study provides insights into and compares the metabolic profiles of the MHO-PCOS and MUO-PCOS phenotypes. The principal aim of this study was to evaluate mtDNA content and oxidation levels between MHO-PCOS and MUO-PCOS women compared to control individuals. Studying mitochondrial parameters in MUO-PCOS and MHO-PCOS individuals is relevant for determining their pathophysiological and clinical characteristics, and potentially informing therapeutic approaches.

## Materials and methods

### Population

#### Women with PCOS and controls

Eighty-one unrelated women with PCOS, aged 16 to 46, were recruited from outpatient clinics at the Endocrine Division of Hospital Carlos G. Durand, Buenos Aires, Argentina. PCOS was diagnosed considering the Rotterdam criteria of 2003 (4). Patients were excluded if any of the following diseases or conditions were reported: history of gestational diabetes, hyperprolactinemia, liver or hematologic disease, Cushing’s syndrome, 21-hydroxylase deficiency, thyroid dysfunction, or diabetes. Women with PCOS were subclassified into MHO-PCOS or MUO-PCOS according to the National Cholesterol Education Program Adult Treatment Panel III criteria (20). Patients with missing data were excluded from phenotypic subclassification.

The control group included 57 unrelated women, aged 18 to 52, recruited from the Hemotherapy Department, Hospital de Clínicas, Buenos Aires, Argentina. To perform a sub-analysis comparing women with PCOS and women with obesity without PCOS, a previously published study (19) was considered. From this study, 38 women with obesity were selected and matched by age, of whom 13 were classified as MHO and 25 as MUO using the ATPIII criteria, which require the presence of at least three out of five components. These include an elevated waist circumference (≥88 cm in women), elevated triglyceride levels (≥150 mg/dL or specific treatment for this condition), reduced HDL cholesterol (<50 mg/dL in women or treatment for this condition), elevated blood pressure (systolic ≥130 mmHg, diastolic ≥85 mmHg, or use of antihypertensive medication), and elevated fasting glucose (≥100 mg/dL or a previous diagnosis of type 2 diabetes). No volunteers had clinical components of PCOS or a family history of PCOS (self-reported), none were taking medication or contraceptives, and all of them had normal clinical parameters. Exclusion criteria included abnormalities in menstrual cycles, visible signs of acne or hirsutism, gestational diabetes, diabetes in first-degree relatives, cardiovascular risk factors, pregnancy, psychiatric history, alcoholism, substance abuse, history or suspicion of pancreatitis, recent corticoid intake, and androgenic alopecia.

The study was conducted in accordance with the Declaration of Helsinki of 1964 and its later amendments or comparable ethical standards and was approved by local institutional research committees at the School of Medicine and the School of Biochemistry of Universidad de Buenos Aires and Hospital Carlos G. Durán, Buenos Aires, Argentina. The patients/participants provided their written informed consent to participate in this study.

### Clinical measures

Anthropometric measures, including height, weight, waist circumference (WC), and body mass index (BMI) were determined using a standardized protocol. WC was measured at the end of normal expiration as the narrowest circumference of the trunk with an inelastic fiberglass standard tape over the bare abdomen, at the narrowest point between the costal margin and the iliac crest. Systolic and diastolic blood pressure (SBP and DBP, respectively) were recorded using a standard mercury sphygmomanometer after at least 10 min of rest. Fasting blood samples were drawn from each individual at 8 a.m. after 12-h overnight fasting during the early follicular phase (days 1-5 of the menstrual cycle for eumenorrheic or oligomenorrheic patients, or at any time for amenorrheic patients). Total cholesterol (TC), triglycerides (TG), low-density lipoprotein cholesterol (LDL-C), high-density lipoprotein cholesterol (HDL-C), glucose, and insulin were measured in serum on a Cobas 8000 autoanalyzer (Roche). Insulin was determined by chemiluminescence on a Liaison instrument (Diasorin) with intra- and inter-assay coefficients of variation of 4.3% and 11.6%, respectively.

The Homeostasis Model Assessment of Insulin Resistance (HOMA index) was calculated using the following formula: (fasting plasma glucose mg/dL x fasting insulin μIU/mL)/405.

To identify patients with PCOS we evaluated in serum the androgen profile, including total testosterone, androstenedione, dehydroepiandrosterone-sulfate (DHEA-S), 17-hydroxyprogesterone (17OHP), sex hormone-binding globulin (SHBG), and the LH/FSH ratio. Total testosterone was measured using a chemiluminescent assay on a Beckman-Coulter Access 2 analyzer, 17OHP and androstenedione were measured using a commercial radioimmunoassay kit (RIA-CT, DIASource), and DHEA-S was determined using an Immulite 1000 kit (DPC). LH and FSH were measured by chemiluminescence on the Access analyzer and SHBG by IMMULITE chemiluminescence. The intra- and inter-assay coefficients of variation were 3.93% and 7.08% for total testosterone, 8.8% and 19.2% for 17OHP, 4.5% and 9.0% for androstenedione, and 9.5% and 15% for DHEA-S. The coefficients of variation for FSH, LH, and SHBG were 7.72%, 6.54%, and 9.93%, respectively. Ovulatory dysfunction was assessed through a review of medical history for menstrual irregularities, such as oligomenorrhea or amenorrhea. Clinical and/or biochemical hyperandrogenism was determined through specific hormonal analyses or documented in the medical history. Polycystic ovarian morphology was identified via transvaginal ultrasound and defined by the presence of ≥12 follicles in each ovary (2–9 mm in diameter) and/or increased ovarian volume (>10 cm³). This diagnostic approach ensured adherence to internationally recognized standards.

### DNA purification and quantification

mtDNA was isolated from peripheral blood leukocytes (CTAB technique) (19).

### Determination of mtDNA content

Two quantitative real-time PCR (qPCR) reactions were performed to amplify an 85 bp fragment of the nuclear gene β2M (β2 microglobulin) and a 108 bp fragment of the mitochondrial sequence MT-TL1. The primers used were 5’CACCCAAGAACAGGGTTTGT3’ (forward) and 5’TGGCCATGGGTATGTTGTTA3’ (reverse) for mtDNA amplification, and 5’TGCTGTCTCCATGTTTGATGTATCT3’ (forward) and 5’TCTCTGCTCCCCACCTCTAAGT3’ (reverse) for β2M (21). The final reagent concentrations were: 3 ng of DNA, 2.5 nM of SYTO 9, 1.5 mM of magnesium chloride, 0.04 mM of dNTPs, 0.75 U of GoTaq DNA polymerase, and 0.5 µM of primers in a final reaction volume of 10 µl. The PCR conditions were: 2 min at 50°C, 20 s at 95°C followed by 40 cycles of 15 s at 95°C, 20 s at 61°C, and 10 s at 72°C, with a melting curve analysis from 50°C to 95°C at a ramp rate of 0.1°C/s, performed on an Applied Biosystems StepOne v2.3 thermocycler. Each sample was analyzed in duplicate, and all measurements included the determination of a positive control, a reference sample, and a negative control without template. Results were calculated using the comparative cycle threshold method. mtDNA content was calculated with the formula: 2x2-^ΔCT^. The control DNA sample was prepared with DNA from peripheral blood leukocytes of 3 women of different ages in equal proportions. The relative mtDNA of each patient was obtained dividing the patient’s mtDNA content by the control sample’s mtDNA content (in percentage) (22).

### Determination of mtDNA oxidation levels

The 8-oxo-guanine residues (8-OxoG) were quantified as a marker of mtDNA oxidation by reactive oxygen species (ROS). The samples were treated with the enzyme formamidopyrimidine DNA glycosylase (FPG; New England Biolabs) according to the manufacturer’s instructions. FPG has N-glycosylase and AP-lyase activity, which release oxidatively damaged purines and generate apurinic sites (AP sites). The AP-lyase activity cleaves each AP site through beta and gamma elimination, creating a single nucleotide gap in the DNA with 5’ and 3’ terminal phosphates. As a result, a delay in the cycle threshold (CT) in qPCR was considered proportional to the level of oxidation as compared to the untreated sample. The previously described mtDNA amplification protocol was used with a primer annealing temperature of 58°C. Results were calculated as ΔCT/mtDNA (%), where ΔCT is the CT difference between the untreated sample and the FPG-treated sample (19).

### Statistical analysis

One-way ANOVA and Tukey *post hoc* tests were used to compare the biochemical, clinical, and anthropometric characteristics, mtDNA content and oxidation level across groups. Independent samples t-test was used to assess mtDNA content and oxidation level between women with PCOS and control women. Linear regression was employed to evaluate the association between mtDNA content, its oxidation levels, and the various biochemical, clinical, anthropometric, and hormonal variables. A univariate general linear model was used to evaluate mtDNA content between the PCOS and control groups, using various covariates: HOMA index, age, BMI, and waist circumference (WC). These variables were introduced into the model separately to assess how each one individually influenced mtDNA content. All statistical analyses were conducted using SPSS v.25 with an age-corrected p-value<0.05. The sample size was calculated using the Sample Size Calculator program as reported in Barrera L. et al. with two independent study groups, PCOS MHO vs. PCOS MUO. Considering a type I (alpha) error of 0.05 (95%), a type II (beta) of 0.05, and a power size of 95%, the minimum number of cases required in the MUO and MHO group was 13 (18).

## Results

### Phenotypic and biochemical characterization of the study population

The anthropometric, clinical, and biochemical characteristics of the PCOS and control groups are presented in **Table 1**. Compared to the control group, women with PCOS displayed significantly higher values in several parameters. Specifically, the PCOS group exhibited increase BMI and WC, as well as elevated TG, LDL-c, total cholesterol, and fasting plasma glucose levels. Furthermore, high- HDL-c levels were markedly lower in the PCOS group. These women also demonstrated significantly higher HOMA-IR values, indicating insulin resistance. Importantly, these differences persisted even after adjustments for age underscoring their robustness.

**Table 1:** Anthropometric, clinical, and biochemical characteristics of the population grouped according to the presence or absence of PCOS.

	Control (n=57)	PCOS (n=81)	p value
Age (years)	30,25 ± 7,21	26,56 ± 5,54	S
WC (cm)	74,27 ± 7,00	96,81± 14,89	S
BMI (kg m^-2^)	21,95 ± 2,10	32,06 ± 7,34	S
Glycemia (mg dl^-1^)	84,04 ± 8,35	88,49 ± 13,17	S
TC (mg dl^-1^)	162,05 ± 33,05	185,37 ± 36,48	S
TG (mg dl^-1^)	68,87 ± 29,32	132 ± 77,21	S
HDL-c (mg dl^-1^)	55,51 ± 14,06	47,9 ± 12,50	S
LDL-c (mg dl^-1^)	91,07 ± 25,59	113,96 ± 29,51	S
SBP (mmHg)	111,67 ± 19,01	112,94 ± 20,71	NS
DBP (mmHg)	74,63 ± 7,66	72,54 ± 11,25	NS
HOMA index	1,65 ± 0,84	3,89 ± 2,90	S

Values are expressed as mean ± SD (test T de Student). PCOS, Polycystic ovary syndrome; WC, waist circumference; BMI, body mass index; TC, total cholesterol; TG, triglycerides; HDL-c, high-density cholesterol; LDL-c, low-density cholesterol; SBP, systolic blood pressure; DBP, diastolic blood pressure; HOMA index, Homeostatic Model Assessment for Insulin Resistance; S, statistically significant, with a p-value< 0.05. NS, Not Significant.

Subsequently, with the aim of evaluating the metabolic impact on mitochondrial parameters, patients with PCOS were stratified into two subgroups using the ATPIII criteria, metabolically healthy obese PCOS (MHO-PCOS) and patients with PCOS and metabolic syndrome (MUO-PCOS). This classification allowed for a more nuanced analysis of how metabolic syndrome influences mitochondrial content and its oxidation level within the PCOS population. The detailed anthropometric, clinical, and biochemical profiles of these subgroups are shown in **Table 2**. Notably, BMI and WC progressively increased across the groups, from controls to MHO-PCOS and then to MUO-PCOS. Patients with MHO-PCOS had significantly higher total cholesterol, LDL-c, and triglycerides compared to controls. They also showed elevated HOMA-IR values, although their blood pressure was lower, and glucose and HDL-c levels were similar to those of the control group. In contrast, patients with MUO-PCOS exhibited significant differences in all measured variables relative to controls, except for blood pressure.

**Table 2:** Anthropometric, clinical, and biochemical characteristics of the population grouped according to metabolic status.

	Groups			Statistical Comparisons			
				*post hoc* Test (Tukey)			
	Control (n=55)	MHO-PCOS(n=44)	MUO-PCOS (n=31)	CTRL vs. MHO-PCOS vs. MUO-PCOS	CTRL vs. MHO-PCOS	CTRL vs. MUO-PCOS	MHO-PCOS vs MUO-PCOS
Age (years)	29,98 ± 7,19	25,55 ± 5,78	28,10 ± 5,09	S	S	NS	NS
WC (cm)	74,27 ± 7,00	92,14 ± 15,91	103,14 ± 10,85	S	S	S	S
BMI (kg m^-2^)	22,00 ± 2,12	29,78 ± 7,50	34,72 ± 6,39	S	S	S	S
Glycemia (mg dL^-1^)	84,06 ± 8,45	85,30 ± 10,25	93,42 ± 15,58	S	NS	S	S
TC (mg dL^-1^)	162,05 ± 33,05	181,57 ± 35,79	190,53 ± 37,97	S	S	S	NS
c-HDL(mg dL^-1)^	55,51 ± 14,06	52,18 ± 13,20	42,00 ± 8,71	S	NS	S	S
c-LDL (mg dL^-1^)	91,07 ± 25,59	109,02 ± 30,37	120,81 ± 27,74	S	S	S	NS
TG (mg dL^-1^)	68,87 ± 29,32	104,95 ± 41,07	174,38 ± 99,44	S	S	S	S
SBP (mmHg)	114,17 ± 11,74	105,00 ± 18,32	122,24 ± 20,25	S	S	NS	S
DBP (mmHg)	74,61 ± 7,75	68,44 ± 10,27	77,24 ± 10,66	S	S	NS	S
HOMA index	1,69 ± 0,84	3,05 ± 2,39	5,15 ± 3,17	S	S	S	S

Values are expressed as mean ± SD (ANOVA and Tukey *post hoc*). MHO-PCOS, Metabolically-healthy with polycystic ovary syndrome women; MUO-PCOS, metabolically unhealthy with polycystic ovary syndrome women; WC, waist circumference; BMI, body mass index; TC, total cholesterol; HDL-c, high-density cholesterol; LDL-c, low-density cholesterol; TG, triglycerides; SBP, systolic blood pressure; DBP, diastolic blood pressure; HOMA index, Homeostatic Model Assessment for Insulin Resistance. S, statistically significant, with a p-value< 0.05. NS, Not Significant.

When comparing MHO-PCOS and MUO-PCOS subgroups, no statistically significant differences in total cholesterol or LDL-c levels were observed. However, MUO-PCOS women had significantly higher glucose, triglycerides, and HOMA-IR values, alongside lower HDL-c and elevated blood pressure compared to MHO-PCOS women. This suggests that MHO-PCOS represents an intermediate metabolic phenotype between the control group and the more metabolically compromised MUO-PCOS group.

### Comparative study of mtDNA content and oxidation level in control, MHO-PCOS, and MUO-PCOS groups

**Figure 1:**
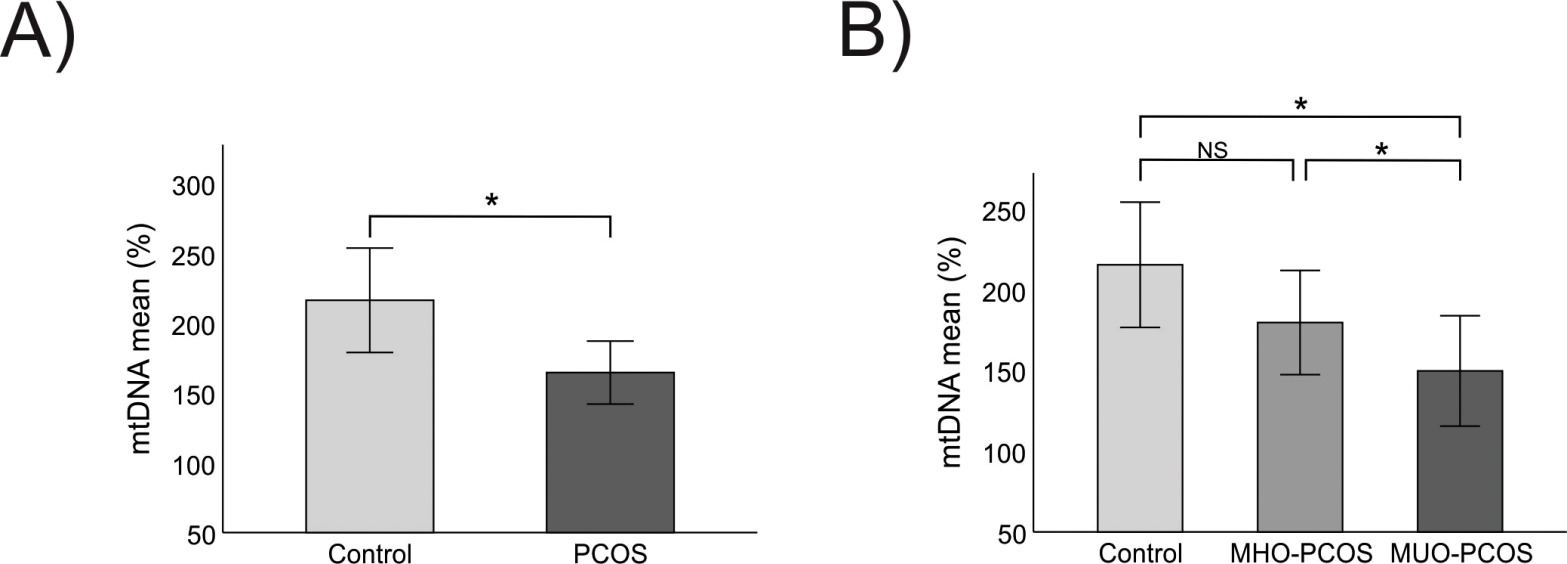
Comparative study of mtDNA content between groups **(A)** Comparison of mtDNA content between control and PCOS group. **(B)** Comparison of mtDNA content between control, MHO-PCOS and MUO-PCOS groups. MHO-PCOS: Metabolically healthy with polycystic ovary syndrome women, MUO-PCOS: metabolically unhealthy with polycystic ovary syndrome women; Comparison of means using Student’s Test, ANOVA and Tukey *post hoc*. *It is considered significant with an age-corrected p-value<0.05. NS, Not Significant.

A comparative analysis of mtDNA content revealed a significant reduction in patients with PCOS compared to control women (164.59 ± 102.07 vs. 215.42 ± 143; p = 0.02). This difference remained statistically significant even after correcting for age (p = 0.04) (**Figure 1A**), WC (p = 0.05), and BMI (p = 0.03), indicating that mtDNA depletion in PCOS is not only driven by these factors. However, when adjusted for HOMA-IR, the differences lost statistical significance, highlighting the significant impact of insulin resistance on mtDNA content.

Further stratification into control, MHO-PCOS, and MUO-PCOS groups revealed a progressive decline in mtDNA content, with mean values of 215.42 ± 143.39 in controls, 179.64 ± 106.12 in MHO-PCOS, and 149.69 ± 93.13 in MUO-PCOS. This gradient emphasizes a potential worsening of mitochondrial parameters as metabolic dysfunction increases, underscoring the impact of metabolic syndrome on mitochondrial parameters. The significant reduction from controls to MUO-PCOS (p = 0.04) suggests a cumulative mitochondrial impairment in more metabolically compromised patients with PCOS. A significant difference emerged between controls and MUO-PCOS women (p = 0.04), which remained significant after age adjustment (p = 0.02) (**Figure 1A**). Additionally, a significant difference was noted between women with MHO-PCOS and with MUO-PCOS (p = 0.05) (**Figure 1B**), underscoring that mitochondrial damage may be aggravated as metabolic syndrome features accumulate.

**Figure 2:**
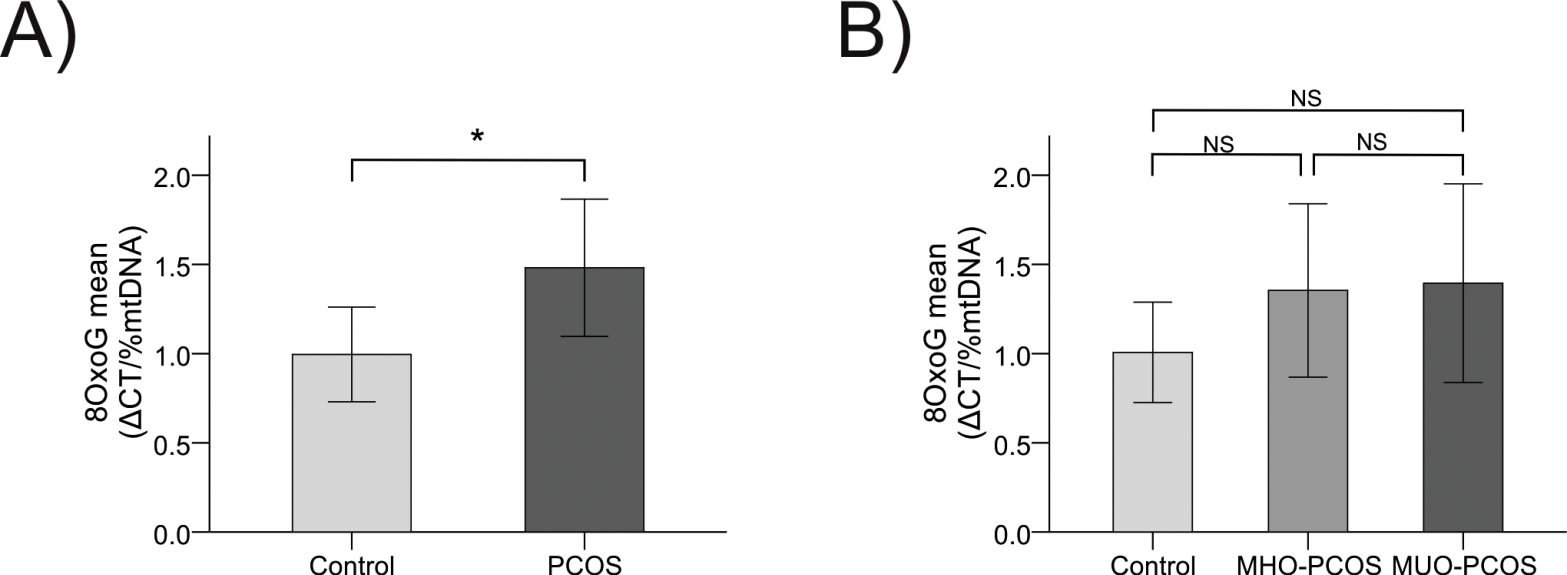
Comparative study of mtDNA oxidation levels between groups **(A)** Comparison of mtDNA oxidation levels between control and PCOS group. **(B)** Comparison of mtDNA oxidation levels between control, MHO-PCOS and MUO-PCOS groups. MHO-PCOS: Metabolically-healthy with polycystic ovary syndrome women, MUO-PCOS: metabolically unhealthy with polycystic ovary syndrome women; Comparison of means using Student’s Test, ANOVA and Tukey *post hoc*. *It is considered significant with an age-corrected p-value<0.05. NS, Not Significant.

Concurrently, patients with PCOS exhibited higher mtDNA oxidation levels than controls (1.48 ± 1.59 vs. 0.99 ± 0.93; p = 0.04). These differences remained significant after correcting for age (p = 0.04) (**Figure 2A**), WC (p = 0.01), and BMI (p = 0.01), though the strength of the association diminished after adjusting for HOMA-IR. An upward trend in mtDNA oxidation was observed across groups, with levels increasing from controls (1.01 ± 0.96) to MHO-PCOS (1.35 ± 1.50) and MUO-PCOS (1.40 ± 1.32), reflecting a possible link between oxidative stress, metabolic worsening and PCOS. However, these trends did not reach statistical significance (**Figure 2B**), suggesting the need for further research to explore the nuances of this relationship. Additionally, we conducted a sub-analysis comparing women with obesity, without PCOS, to women with PCOS from the current study. As shown in the **Supplementary Figure 1**, women with PCOS, regardless of metabolic health status, exhibited lower mtDNA content and higher oxidation levels than obese women without PCOS. The most severe alterations were observed in MUO-PCOS, suggesting that PCOS exacerbates mitochondrial dysfunction, especially in the presence of MS.

### Associations between anthropometric markers, insulin resistance, and mitochondrial parameters

**Figure 3:**
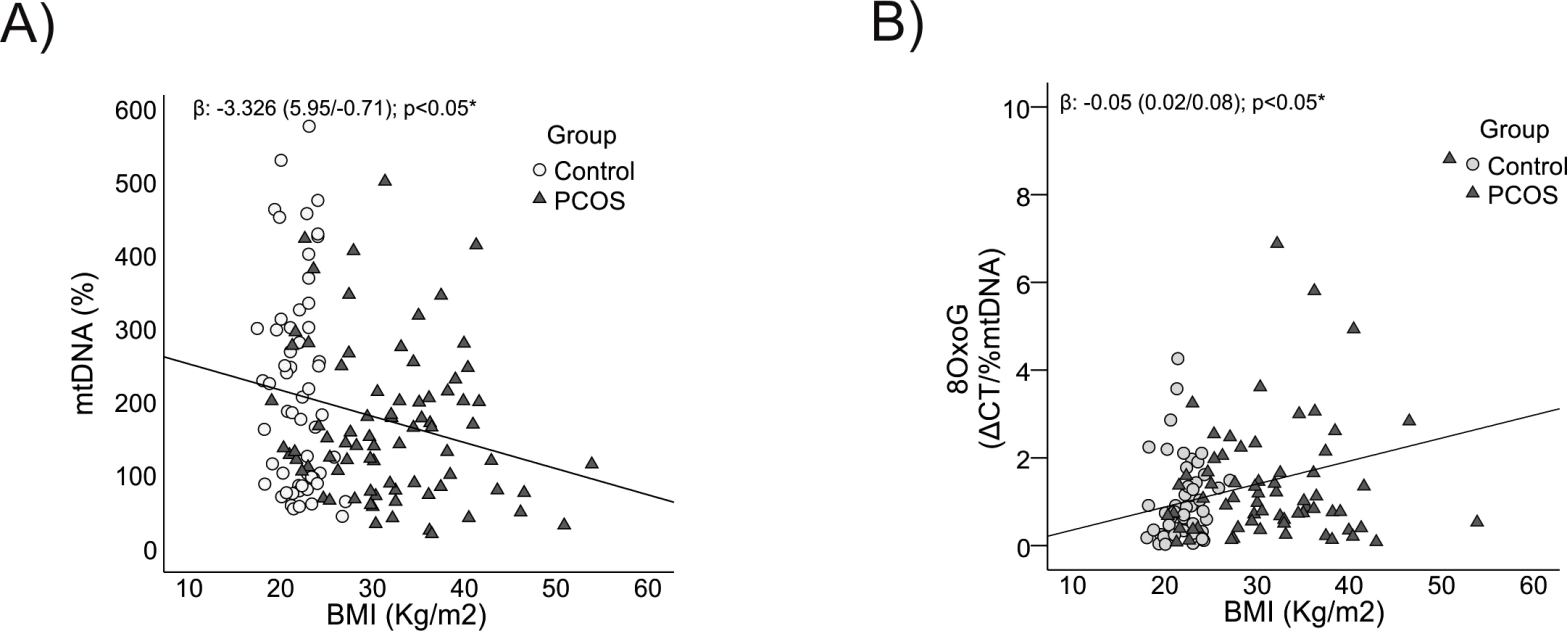
Relationship between mtDNA content and its oxidation level with BMI. **(A)** With mtDNA content. **(B)** With 8-OxoG. BMI: body mass index, *It is considered significant with an age-corrected p-value<0.05.

**Figure 4:**
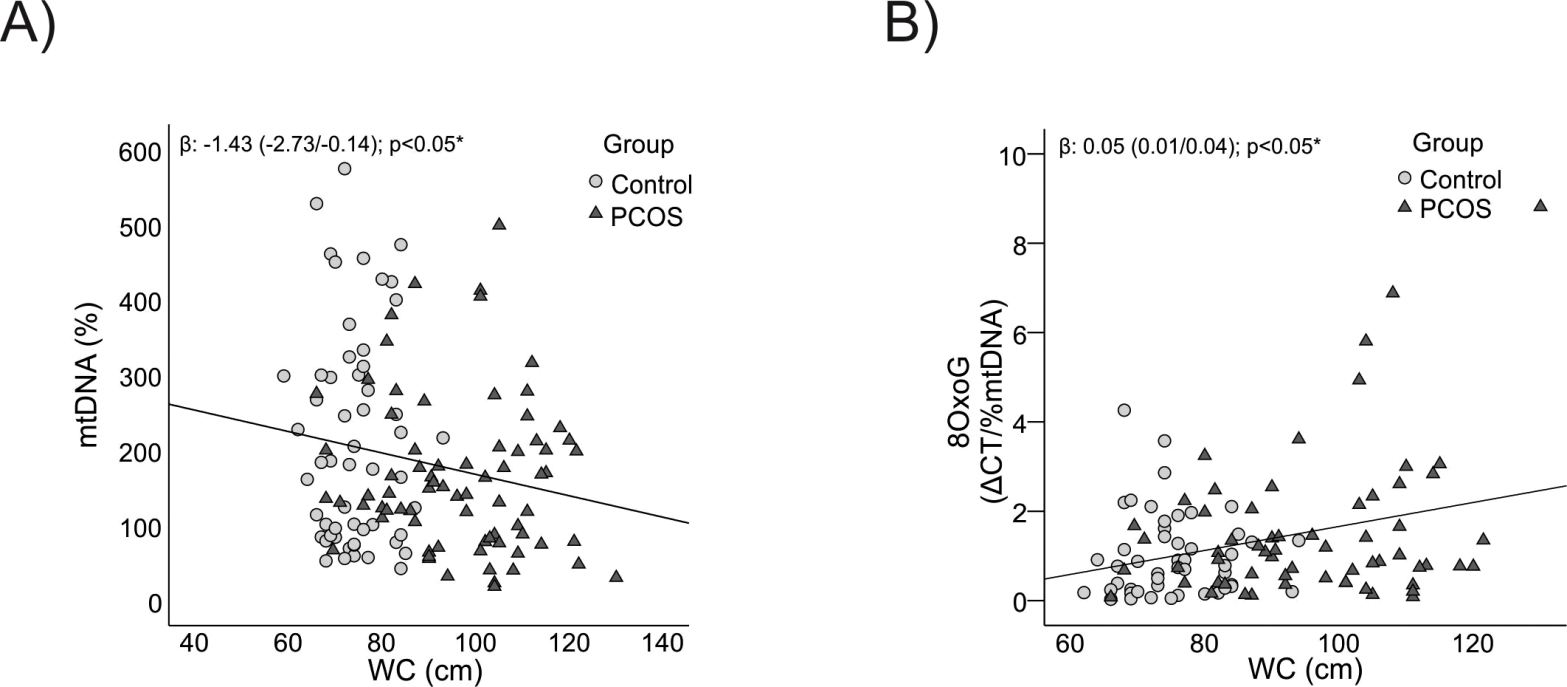
Relationship between mtDNA content and its oxidation level with WC. **(A)** With mtDNA content. **(B)** With 8-OxoG. WC: waist circumference, *It is considered significant with an age-corrected p-value<0.05.

Analyzing the associations between mitochondrial parameters and anthropometric markers revealed a clear pattern: mtDNA content showed a significant negative correlation with both BMI (p = 0.01) and WC (p = 0.03) (**Figures 3A**, **4A** respectively), indicating that higher body mass and central adiposity are associated with a reduction in mtDNA content.

Conversely, mtDNA oxidation levels were positively correlated with BMI and WC (p = 0.01 for both) (**Figures 3B**, **4B** respectively), highlighting that greater adiposity is linked to elevated oxidative stress. This relationship underscores the role of obesity and central fat accumulation in driving oxidative damage, which could further impair mitochondrial function and exacerbate metabolic disturbances.

**Figure 5:**
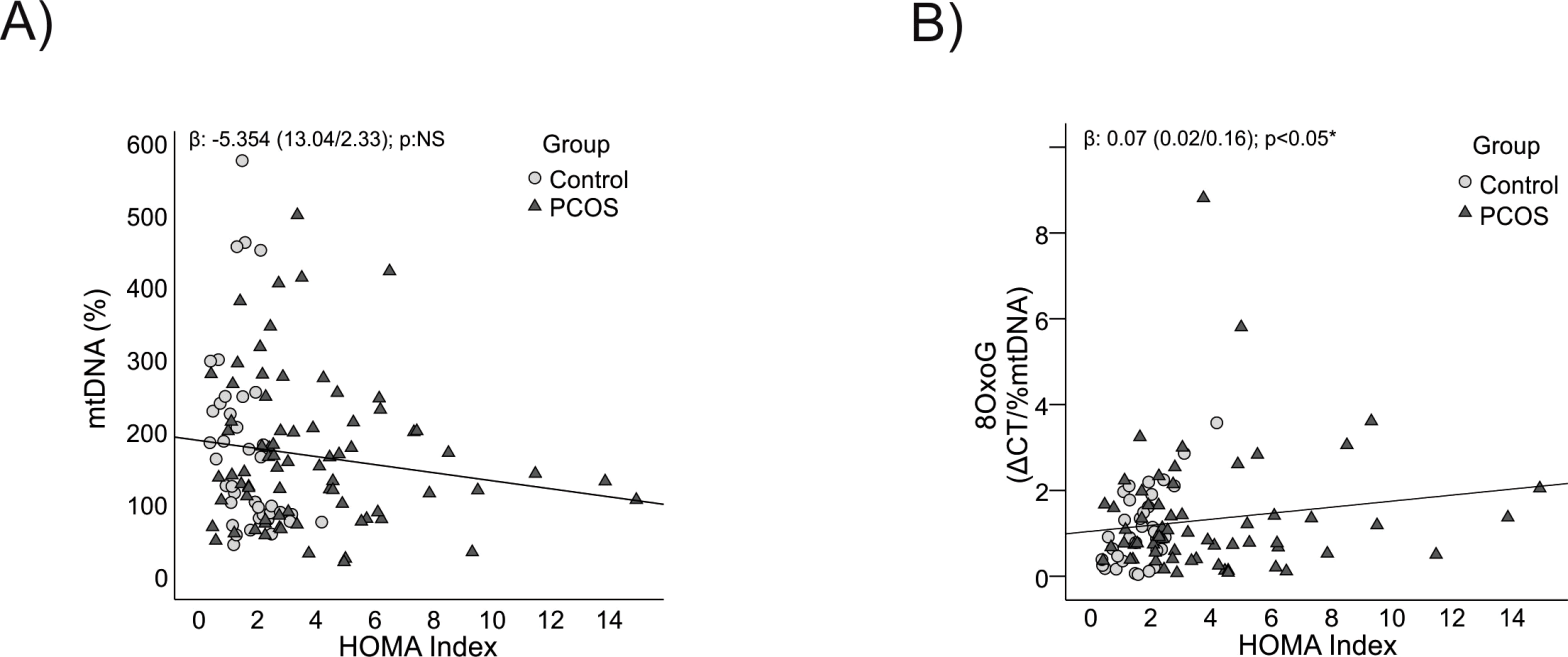
Relationship between mtDNA content and its oxidation level with insulin resistance. **(A)** With mtDNA content. **(B)** With 8-OxoG. HOMA index: Homeostatic Model Assessment for Insulin Resistance, *It is considered significant with an age-corrected p-value<0.05. NS, Not Significant.

Furthermore, as the HOMA index, a measure of insulin resistance, increased, there was a trend towards decreased mtDNA content (p = 0.08) (**Figure 5A**), although this did not reach statistical significance. However, mtDNA oxidation levels showed a significant increase after adjusting for age (p = 0.02) (**Figure 5B**), emphasizing the substantial impact of insulin resistance on oxidative stress. These findings suggest a dual impact where higher adiposity and insulin resistance contribute both to mtDNA depletion and its oxidative damage, which may worsen metabolic outcomes in PCOS.

## Discussion

This study provides insights into the metabolic profile of patients with PCOS as compared to control women and analyzes the MUO-PCOS and MHO-PCOS phenotypes. Even though few studies have so far explored the clinical relevance of the MHO and MUO phenotypes among obese PCOS women, our findings on the differences in metabolic risk profiles in adult patients with PCOS agree with previous research (13–16).

Despite the limited data on mtDNA content in MHO and MUO women with PCOS, mtDNA could be considered a relative indicator of mitochondrial health (23). Indeed, the decline in β-oxidation observed in mitochondrial dysfunction causes electron leakage toward oxygen and the formation of O_2_, which generates ROS and leads to oxidative damage to all macromolecules, including mtDNA (24). Previous reports have shown that the number of mtDNA copies is significantly smaller in women with PCOS and negatively correlates with ROS levels (25–27), which reduces cellular metabolic activity (28) and triggers mitochondrial damage or dysfunction (29). Along these lines, our results showed lower mtDNA content and higher oxidation levels in patients with PCOS as compared to control women.

Several studies have demonstrated a close association between elevated ROS levels and the development of PCOS (30); the relationship between obesity, PCOS, and metabolic disease, however, remains unclear. In this study, a progressive decrease in mtDNA content was observed from the control group to the MHO-PCOS and MUO-PCOS groups indicating that lower mtDNA content was associated with obesity and a worse metabolic profile. Based on the sub-analysis conducted, our findings indicate that women with PCOS have lower mtDNA content and higher oxidative damage compared to obese women without PCOS, suggesting that PCOS exacerbates mitochondrial dysfunction beyond the effects of obesity. It is important to emphasize that these results are derived from different cohorts. New recruitment efforts, including these specific subgroups of patients, will be necessary to confirm this claim and draw definitive conclusions regarding these observations.

Alterations in anthropometric parameters, such as BMI and WC, show a significant relationship with mtDNA content and its oxidation levels due to their direct impact on metabolic profile and body fat distribution. While BMI reflects overall excess weight, WC provides information on abdominal and visceral fat accumulation, which is more closely associated with the risk of insulin resistance, oxidative stress, and chronic inflammation. These metabolic factors, particularly visceral fat, are known to contribute to mitochondrial dysfunction, which explains the negative correlation with mtDNA content and positive correlation with oxidation levels in the context of PCOS.

PCOS is associated with factors that may contribute to oxidative stress, such as chronic inflammation, insulin resistance, and hormonal imbalance. The increased levels of oxidation in women with PCOS, despite no differences between groups with or without metabolic syndrome, reinforce the idea that the pathophysiological characteristics of PCOS directly affect mitochondrial function, leading to mtDNA oxidation independently of metabolic status.

Insulin Resistance is a common characteristic in PCOS, Lee and colleagues showed that in women with PCOS, mtDNA content correlated negatively with IR (28). In this study, we observed a trend toward to lower mtDNA content when HOMA index increased, and a positive correlation between this parameter and mtDNA oxidation levels. It should be noted that mitochondrial dysfunction is known to lead to IR. More specifically, mitochondrial dysfunction promotes a decrease in lipid oxidation due to a general decrease in oxidative phosphorylation efficiency; this in turn contributes to the accumulation of free fatty acids (FFAs) and lipids -together with an increase in the levels of diacylglycerides (DG) and ceramides-, all of which inhibit insulin signaling (31).

Some studies have reported a smaller number of mtDNA copies in the peripheral blood of patients with PCOS as compared to the control group regardless of IR or other metabolic factors (28). In our study, however, the decline in mtDNA content and the increase in oxidation levels between controls and patients with PCOS lost significance when these relationships were adjusted for the HOMA index. This finding suggests that IR could be the main factor contributing to mitochondrial dysfunction in PCOS, supporting the hypothesis that IR plays a key role in the mitochondrial changes observed in these patients. However, it cannot be ruled out that there may be other underlying causes not addressed in this study. Determining the independent contributions of PCOS and metabolic syndrome on mitochondrial parameters is a limitation of this study. Future research that includes populations of obese patients without PCOS, both with and without metabolic syndrome, will be valuable to enhance the understanding of metabolic status-specific differences associated with PCOS.

These findings could have important clinical implications, as if PCOS itself contributes to oxidative damage, it could justify the need for specific interventions that address oxidative stress in patients with PCOS, regardless of their metabolic profile. This could include approaches to reduce inflammation and oxidative stress. Given that women with PCOS have an increased risk of developing long-term metabolic diseases, such as type 2 diabetes and cardiovascular diseases, continuous monitoring of oxidative damage and mtDNA content could be essential for assessing the risk of these comorbidities. While these findings are promising, their generalization requires further studies in cohorts that include other ethnic groups, due to their genetic and environmental heterogeneity.

## Conclusion

The results of this study show that women with PCOS exhibit decreased mtDNA content and increased oxidation levels compared to control women, indicating underlying mitochondrial dysfunction. This dysfunction correlates with a progressive increase in WC and significantly influences IR, suggesting mtDNA content plays a role in develop of IR in patients with PCOS. When assessing mtDNA, we found that metabolic factors contribute more significantly than PCOS itself. However, when analyzing mtDNA oxidation levels, PCOS appears to have a greater influence. In both parameters, insulin resistance shows a notable impact. Further research is needed to better understand the specific contributions of each factor to mitochondrial parameters. The findings of this study highlight the importance of maintaining adequate mtDNA copies for preserving mitochondrial function. The observed reduction in mtDNA content and increased oxidation levels in women with PCOS enhance our understanding of the relationship between mitochondrial dysfunction, obesity, and PCOS. These findings could have significant clinical implications, suggesting the need for interventions targeting oxidative stress in patients with PCOS. However, further studies are necessary to assess their broader applicability.

## Data Availability

The raw data supporting the conclusions of this article will be made available by the authors, without undue reservation.
