# Supplementary figures and images for: Mitochondrial DNA oxidation and content in different metabolic phenotypes of women with polycystic ovary syndrome
Source: Front Endocrinol (Lausanne). 2025 Jan 9;15:1501306. doi: 10.3389/fendo.2024.1501306 (PMC11754062; doi:10.3389/fendo.2024.1501306)

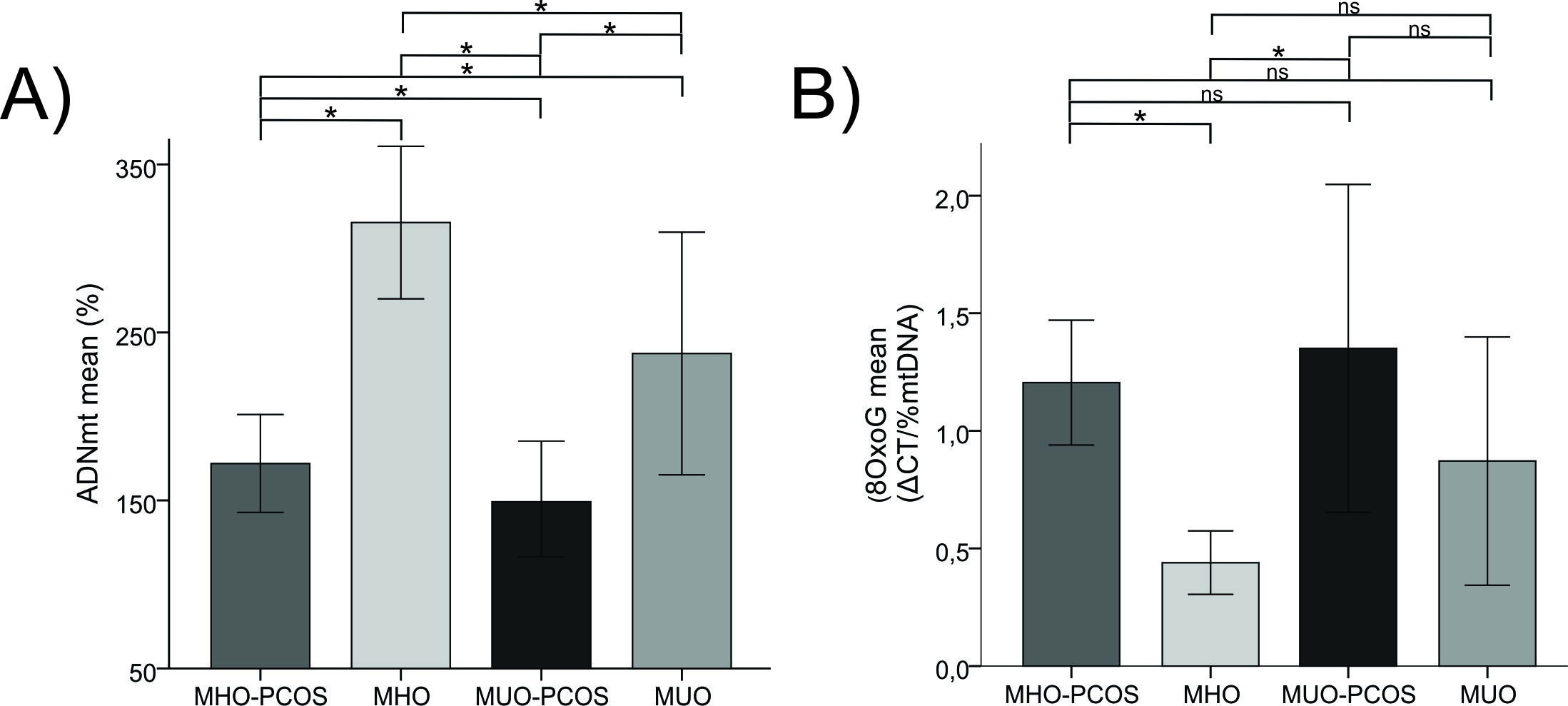

Supplement: Supplementary file 1 [file Image1.jpeg]
